# Neural activity in relation to empirically derived personality syndromes in depression using a psychodynamic fMRI paradigm

**DOI:** 10.3389/fnhum.2013.00812

**Published:** 2013-12-04

**Authors:** Svenja Taubner, Daniel Wiswede, Henrik Kessler

**Affiliations:** ^1^Hanse Institute for Advanced StudyDelmenhorst, Germany; ^2^Department for Psychology, Alpen-Adria-Universität KlagenfurtKlagenfurt, Austria; ^3^Department of Neurology, University of LübeckLübeck, Germany; ^4^Department of Psychosomatic Medicine and Psychotherapy, LWL University Hospital, Ruhr-University BochumBochum, Germany

**Keywords:** depression, psychodynamic diagnosis, fMRI, Shedler–Westen Assessment Procedure, personality syndrome

## Abstract

**Objective:** The heterogeneity between patients with depression cannot be captured adequately with existing descriptive systems of diagnosis and neurobiological models of depression. Furthermore, considering the highly individual nature of depression, the application of general stimuli in past research efforts may not capture the essence of the disorder. This study aims to identify subtypes of depression by using empirically derived personality syndromes, and to explore neural correlates of the derived personality syndromes.

**Materials and Methods:** In the present exploratory study, an individually tailored and psychodynamically based functional magnetic resonance imaging paradigm using dysfunctional relationship patterns was presented to 20 chronically depressed patients. Results from the Shedler–Westen Assessment Procedure (SWAP-200) were analyzed by Q-factor analysis to identify clinically relevant subgroups of depression and related brain activation.

**Results:** The principle component analysis of SWAP-200 items from all 20 patients lead to a two-factor solution: “Depressive Personality” and “Emotional-Hostile-Externalizing Personality.” Both factors were used in a whole-brain correlational analysis but only the second factor yielded significant positive correlations in four regions: a large cluster in the right orbitofrontal cortex (OFC), the left ventral striatum, a small cluster in the left temporal pole, and another small cluster in the right middle frontal gyrus.

**Discussion:** The degree to which patients with depression score high on the factor “Emotional-Hostile-Externalizing Personality” correlated with relatively higher activity in three key areas involved in emotion processing, evaluation of reward/punishment, negative cognitions, depressive pathology, and social knowledge (OFC, ventral striatum, temporal pole). Results may contribute to an alternative description of neural correlates of depression showing differential brain activation dependent on the extent of specific personality syndromes in depression.

## INTRODUCTION

According to the WHO depression is one of the most prevalent diseases worldwide (World Health Organization, 2002) that goes along with substantial symptom severity and role impairment (Kessler et al., 2003) and is therefore a major public health issue. The distinction between different forms of chronic depression in the DSM-IV (Diagnostic and Statistical Manual of Mental Disorders, Fourth Edition) has recently been criticized because patients with dysthymia, double depression, or major depressive disorders seem to have only minor differences in their clinical features, family history, and treatment response (McCullough et al., 2000, 2003; Klein et al., 2004). On the other hand, there is obvious heterogeneity between patients with depression, which is clinically relevant but cannot be captured adequately with existing descriptive systems of diagnosis (DSM-IV, APA, 1994; ICD-10, WHO, 1992). As for the idea of “clinical relevance,” subtyping depression is not only important for the sake of taxonomic clarity. Dwelling into the complexities of depression by garnering a more nuanced picture of the disorder might also facilitate case conceptualization and treatment planning for clinicians. Relevant in the context of our study, a differentiated picture of depression and its neurobiological underpinnings examined by brain imaging might eventually lead to different therapeutic approaches or be useful as a predictor for relapses.

Among other important approaches to define depression and its subtypes, Bleichmar (2010) describes different pathways of pathological mourning which is related to chronic depression and factors of maintaining depression. He suggests that the essential component of pathological mourning is the feelings of helplessness and hopelessness related to the loss of a significant other or a feature of a significant other (e.g., love). Bleichmar (2010) distinguishes at least two subtypes in pathological mourning: while the first subtype is related to a past loss, patients of the second subtype are suffering from a current loss of internal or external causes (e.g., loss of employment). Especially the second subtype is characterized by relationship anxiety and hostility toward others which often isolates them from corrective or helpful relationship experiences. Aggressiveness and ambivalence toward others as a certain subtype of depression has also been described by Freud (1917) in “Mourning and Melancholia” and was confirmed in psychoanalytic clinical work by Klein (1940) and Jacobson (1971). According to Bleichmar (2010), understanding the complex interaction of etiology and maintaining factors of depression is important to establish individually tailored treatment modalities. In Bleichmar’s view, subtypes of depression are best understood within a dimensional model of the psyche, which resembles the psychoanalytic approach to nosology in contrast to approaches using isolated categories, such as the DSM. Regarding another alternative approach to differentiate within the spectrum of patients with depression that stands in contrast to the DSM-IV typology of depression, Blatt and Luyten (2009) have suggested to distinguish between introjective and anaclitic depression (for a more basic criticism on the DSM-IV and depression compare Luyten et al., 2006). The anaclitic depression is based on feelings of loneliness, neglect, abandonment, and staying in relationships whereas the introjective depression is centered on self-worth, failure, guilt, and a withdrawal from relationships. The introjective pole has been related to chronic depression and poorer treatment outcome (Blatt et al., 2001; Blatt, 2004, 2008). Tackling the issue of heterogeneity in depression and using a diagnostic method closer to clinical inference, Westen and Shedler (1999b) and Shedler and Westen (2004) derived five empirical subtypes of depression relating to different triggers for depressive moods: (a) avoidant, (b) high-functioning, (c) dysregulated, (d) dependent, and (e) hostile-externalizing.

In general, complex clinical inferences are based on a variety of psychological data including not only what a patient says but how something is said as well as how this affects the clinician emotionally (Westen and Arkowitz-Westen, 1998). This way of thinking and inferring is the core of psychodynamic understanding (Kernberg, 1975; McWilliams, 1994) which is in contrast to rather technical diagnoses that list symptoms without relating them to each other. In a psychodynamic approach, clinicians do not count symptoms but compare an individual patient with a prototype of the disorder (Blashfield et al., 1985; Kim and Ahn, 2002). However, clinical diagnoses are found not to be reliable and are therefore considered not being useful in empirical research. The Shedler–Westen Assessment Procedure (SWAP-200; Shedler and Westen, 2007; Westen et al., 2011) tries to bridge the gap between clinical practice and empirical research by providing a diagnostic tool that relies on clinical judgment with a standardized vocabulary and Q-sort method to obtain meaningful data on personality pathology independent from theoretical approach. Hence, the SWAP-200 is a theoretically and empirically well-grounded method to encounter the phenomenological heterogeneity we face on the clinical side of depression.

The clinical heterogeneity in depression is comparably evident from the neurobiological perspective. Over the last 15 years, many studies with increasing sophistication and reviews could narrow down the brain areas presumably involved in the pathophysiology of depression [amygdala, basal ganglia, prefrontal cortex, anterior cingulate cortex (ACC), etc.] but there is still no consensus regarding for instance the hemisphere in which these changes are most prominent or the exact direction of the differences in activation (Drevets, 2000; Davidson et al., 2002; Mayberg, 2003; Fitzgerald et al., 2006; Steele et al., 2007). A comprehensive meta-analysis found only limited overlap between studies exploring brain changes in depression: prefrontal cortex, ACC, insula, and superior temporal gyrus were found to be relatively hypoactive, whereas several limbic, subcortical, and frontal regions showed hyperactivity (Fitzgerald et al., 2008). Besides the above mentioned structures, a recent meta-analysis stresses the importance of an increased pulvinar nucleus baseline activity in patients with depression, which increases the responsiveness of the salience network and hinders the prefrontal structures from reappraisal (Hamilton et al., 2012). However, considering those data the heterogeneity of the neural correlates of depression prevails. There are various methodological reasons for the problems we encounter with neuroimaging data (Gusnard et al., 2001; Logothetis, 2008; Kriegeskorte et al., 2009; Vul et al., 2009). In addition to the heterogeneity of patient samples that also plagues neurobiological studies, one central problem could be the mere application of generalized stimuli in the vast majority of neuroscientific studies in the field. Considering the highly individual nature of depression in terms of history, relationship patterns, personality functioning, and others, merely applying experiments with general stimuli (e.g., emotional faces, Ekman and Friesen, 1976; or pictures from the International Affective Picture System, Lang et al., 1997) hardly captures the essence of the disorder. Consequently, the individualization of experimental paradigms could tackle the issue of heterogeneity in depression as well as in its neurobiological underpinnings. It is only through a differentiated and individualized approach that we could adequately assess the phenomenon of depression in its nuances. Amongst other authors, this has been clearly stated by Kessler et al. (2011b) for the case of the neurobiology of depression and recently for the investigation of neural correlates of changes after psychodynamic psychotherapy (Böker et al., 2013). For the clinical side, concepts like the SWAP-200 contribute to a more nuanced and differentiated view of each patient’s depression. In an endeavor to account for both aspects described above, our study presented here uses a laborious but rich description of depression subtypes by deriving dimensional personality syndromes with the SWAP-200 in combination with a complex experimental functional magnetic resonance imaging (fMRI) paradigm applying individualized stimuli. In our opinion, individualization is the pivotal aspect when investigation the neurobiology of depression. We hence developed our own set of stimuli consisting of sentences describing each patient’s dysfunctional relationship pattern and psychodynamic conflict-related themes (Kessler et al., 2011a). The sentences were derived from a clinical interview based on operationalized psychodynamic diagnosis (OPD; OPD-Task-Force, 2008) and were suitable for presentation in the fMRI scanner (see Materials and Methods for details).

Patients were confronted with their individualized psychodynamic relation themes in the fMRI scanner to increase the impact and specificity of brain responses. Part of the data of this experiment was already presented (Kessler et al., 2011a). This report adds a rich clinical aspect: in addition, two interviews with patients were used for the assessment of the SWAP-200 to obtain data on personality functioning in terms of a dimensional approach which is in line with the research suggestions from DSM-V (Skodol et al., 2011). Based on this assessment a Q-factor analysis extracted two factors describing meaningful clinical personality phenomenology. Patients’ scores on the spectrum of those extracted factors were then correlated with relative brain responses to the OPD stimuli. Since the exact amount and nature of the SWAP-200 factors were unknown before data collection, we did not follow any specific hypotheses. The main study question was hence on a more exploratory level if personality syndromes in depression correlate with brain activity during a depression-related and individualized experiment.

## MATERIALS AND METHODS

### PARTICIPANTS

Participants comprised 20 unmedicated patients [age *M* (SD) = 39.2 years (12.7), range 20–64 years, 16 women] with recurrent major depressive disorder. All patients were in a major depressive episode during recruitment. Data of 18 of those patients have been included in a previous report comparing patients with controls (Kessler et al., 2011a). Patients were recruited from a psychoanalytic institute and diagnosed by two trained clinicians using the structured clinical interviews I and II for DSM-IV (SCID; German version; Wittchen et al., 1996). They reported between 1 and 10 depressive episodes [*M* (SD) = 4.00 (3.58)], and their age at first occurrence of depression was between 8 and 40 years [*M* (SD) = 20.00 (9.52)]. Some patients had received various types of medication and psychotherapies during the course of their disease but had not received treatment within at least 6 months prior to inclusion in the study. After study inclusion and baseline assessments all patients started a psychoanalytic psychotherapy. Exclusion criteria were other psychiatric conditions, substance abuse, significant medical or neurological conditions, or eye problems. The study protocol was approved by the ethics committee of the University of Ulm.

### CLINICAL MEASURE

The SWAP-200 is a Q-Sort procedure with 200 clinical statements that have to be sorted in a fixed distribution (Westen and Shedler, 1999a,b). Items were drawn from the clinical literature of the past 50 years, research literature on coping, defense and affect regulation, interpersonal pathology, and personality research in non-clinical populations. Each item may describe a patient well, a little or not at all. Coders sort all items into a fixed distribution, ranking from most descriptive (value 7) to least descriptive (value 0). The SWAP-200 is based on a Q-Sort-method which forces the coder to rank the 200 items in a fixed distribution. The instrument is available online ().

Items are constructed jargon-free and if possible close to observation, e.g., “tends to abuse alcohol,” “tends to have numerous sexual involvements; is promiscuous.” Statements that describe psychic processes, that have to be inferred from the interview situation or descriptions from the patients, are constructed in clear unambiguous language, e.g., “tends to be conflicted about authority (e.g., may feel s/he must submit, rebel against, win over, defeat, etc.)” or “appears to have little need for human company or contact; is genuinely indifferent to the presence of others.”

The SWAP-200 shows high inter-rater reliability between *r* = 0.80 and 0.90 (Shedler and Westen, 1998; Westen and Muderrisoglu, 2003; Marin-Avellan et al., 2005; Westen and Shedler, 2007). It has been validated on 797 US-American psychotherapists of different therapeutic approaches of whom 72.7% concluded that the SWAP-200 allows them to describe the most important aspects of their patients (Westen and Shedler, 1999a); convergent and discriminant validity was also confirmed (Westen and Shedler, 1999a,b; Cogan and Porcerelli, 2005).

Although it is recommended to score the SWAP-200 using the Clinical Diagnostic Interview (Westen, 2002), it is also possible to use it with other diagnostic interviews or on the basis of at least five therapeutic sessions (Westen and Weinberger, 2004). In the present study, the SWAP-200 was scored by two clinical psychologists in a consensus rating on the basis of two video- and audiotaped clinical interviews with each participant: the Scales of Psychological Capacities Interview (SPC; Huber et al., 2006a,b) and an interview based on the OPD (OPD-Task-Force, 2008).

### FACTOR ANALYSIS

We applied Q-factor analysis to identify personality syndromes empirically. Q-factor analysis enables groupings of patients with personality features similar to one another and distinct from those of patients in other groupings. The statistical procedure is identical with conventional factor analysis but is applied to cases rather than variables. Therefore, our data matrix was transposed, using cases as variables (columns) and SWAP-200 items as cases (lines) (cp. Block, 1978; Westen et al., 2011). This leads to 200 “cases” with 20 variables each which is sufficient to conduct a principle component analysis. Classical factor analysis identifies groups of similar variables that belong to a common underlying factor. In contrast, Q-factor analysis identifies groups of similar people who share characteristics, in this case common personality syndromes. The findings reported here are based on a principle component analysis without rotation. We decided against rotation in terms of a “simple structure approach” due to theoretical reasons because we expected to find a common personality factor as well as factors that differentiate between patients (Russell, 2002). Using a varimax rotation, for example, would force a solution with two or more orthogonal factors. Since all patients were diagnosed with depression, we expected that all participants would load on one factor related to depression but differ in their factor loadings on other factors. Therefore, using a principle component analysis without rotation would allow analyzing factors that are closer to the clinical phenomenon of depression. Statistical analysis was performed using the Statistical Package for the Social Science (SPSS) version 19.0. After identifying factors with principle component analysis, we analyzed those 20 items from the SWAP-200 loading highest on each factor. Descriptive core features of each factor dimension were summarized and interpreted by two clinicians (Henrik Kessler and Svenja Taubner) to obtain a diagnostic description of each factor.

### STIMULI, PROCEDURE, AND EXPERIMENT

Individualized stimuli were generated based on an interview according to the system of OPD (OPD-Task-Force, 2008) conducted by a trained clinician (Henrik Kessler). Videotaped material was rated independently by two to three expert raters (OPD-trainers). Typical dysfunctional interpersonal relations were identified and served as basis for the experimental stimuli (“OPD sentences”). Sentences described an individual problematic interpersonal relation typical of their depressive cognitions. Four individually tailored sentences were selected for each participant representing the typical dysfunctional relationship theme of each person (e.g., “You wish to be accepted by others.”, “Therefore you do a lot for them.”, “That is often too close for them, so they retreat.”, “Then you feel empty and lonesome.”). These individual sentences served as stimuli during the fMRI session (experimental or OPD condition). The control condition (“traffic”) comprised four sentences, which described a stressful traffic situation (“The other driver makes a mistake.”, “You are very upset about this.”, “You react to the other driver.”, “But he reacts inadequately.”). Prior to testing, participants were asked to remember a recent and stressful situation they had experienced in traffic. The rationale behind this control condition was to induce negative emotions and recall autobiographical memories including human interactions, but without engaging in specific depression-related material. In order to separate the two conditions (OPD and traffic), and let subjects calm down after emotionally demanding sentences, “relaxation” sentences were inserted between conditions. Those sentences instructed participants to relax. Whereas the OPD sentences were derived individually for each person, “relaxation” and “traffic” were the same across all subjects. OPD sentences were slightly but significantly longer [*M* (SD) = 50.8 (8.0) characters] than “traffic” sentences (44 characters, *p* < 0.001).

Four to six weeks prior to the fMRI assessment, participants filled out informed consent forms and were interviewed (SCID I+II, OPD, SPC). At the beginning of the fMRI session, they were briefed, saw their individual OPD sentences prior to actual scanning and were asked, whether the sentences fit and enticed them to think about their problematic relations. After completion of assessments, all patients started psychodynamic treatment.

### IMAGE ACQUISITION AND ANALYSIS

Sentences were presented by a projector onto a screen watched by the participants via a mirror while lying in the scanner. The four sentences of a condition (OPD, traffic, relaxation) were presented for 7.5 s each, resulting in 30 s blocks. During the OPD block participants were asked to mentally engage in situations with significant others, as described by the OPD sentences. They received no instruction to regulate their emotions, but were asked to let spontaneous thoughts, emotions, and memories come to mind. “Traffic” and “relaxation” conditions also comprised four sentences with each lasting 7.5 s. The instructions were to mentally engage either in the traffic situation or to relax. In total, 12 “relaxation,” 6 “traffic,” and 6 “OPD” blocks were presented (white Arial font, size 16, black background). Blocks were separated by a 5-s fixation cross. The entire experiment lasted approximately 15 min.

Data were obtained using a 3 T SIEMENS Magnetom Allegra head scanner (Siemens, Erlangen, Germany). Participants were positioned on the scanner couch and wore foam earplugs to reduce scanner noise. An experienced psychotherapist not involved in the therapy of the patients (Svenja Taubner or Henrik Kessler) assisted with the setup procedure and coached the participants throughout the experiment. Data acquisition started with anatomical images (3D high resolution T1-weighted isotropic volume, MPRAGE-sequence [MPRAGE = Magnetization Prepared Rapid Gradient Echo (18)]; repetition time (TR) = 2.3 s, field of view (FOV) = 256 mm × 256 mm × 176 mm, echo time (TE) = 4.38 ms, inversion time (TI) = 900 ms, flip angle = 8°, 1 mm isovoxel, total acquisition time 14.45 min). Functional scans were performed using a single shot echo planar imaging (EPI) sequence. A total of 365 T2*-weighted whole-brain volumes were acquired (EPI-sequence; TR = 2500 ms, TE = 30 ms, flip angle = 90°, FOV = 192 mm, matrix 64 × 64, 44 slices, slice thickness 3 mm, interleaved acquisition order, AC–PC (anterior commissure–posterior commissure) orientation, total acquisition time: 15.18 min).

Data were analyzed and visualized using Brain Voyager QX 1.10 to 2.2 (Brain Innovation, Maastricht, Netherlands). Preprocessing: functional data were slice-time corrected and motion was corrected relative to the first volume of the run. To remove low frequency drifts, data were high-pass filtered (three cycles, three sine waves fall within the extent of the data). Structural and functional data were transformed into the standard space of Talairach and Tournoux, data points were labeled using Talairach Daemon. The design matrix was modeled using the two gamma hemodynamic response function. Functional data were smoothed using an 8-mm full width at half maximum (FWHM) isotropic Gaussian kernel. A random effects analysis based on *z*-transformed functional data was conducted including the within-factor CONDITION (OPD vs. traffic sentences). Motion-correction parameters were included in the generalized linear model (GLM) as regressors of no interest.

Whole-brain correlational analyses were conducted based on individual values within the SWAP-200 factors extracted by Q-factor analysis and beta values for the contrast OPD > traffic for all subjects. Whole-brain statistics were conducted and maps are shown with a threshold of *p* < 0.001, uncorrected. A cluster size threshold of 16 voxels was consistently applied. All active voxels are displayed in native resolution without interpolation and plotted on the Talairach-transformed brain; Talairach coordinates are reported as TAL *x*, *y*, *z*.

## RESULTS

### SWAP-200 FACTOR ANALYSIS

The principle component analysis of SWAP-200 items from all 20 patients lead to a two-factor solution (eigenvalues: 9.30, 1.55) accounting for 54.23% of the variance, Factor 2 explained 7.8% and Factor 1 explained 46.5% of the variance.

The 20 items with the highest factor loadings on Factor 1 could be summarized as the following: one set of items described depressive symptoms (Items 1, 3, 4, 5, 6, 10, and 11, compare **Table 1**), another set of items resembled relationship problems and relationship anxieties typical for depressed patients, e.g., the inhibition or questioning of own wishes and problems in expressing anger (items 2, 7, 9, 12, 14, 15, 16, 17, 18). Two items could be interpreted as adding a momentum of self-criticism to the factor (8, 13). Because self-criticism was only represented by two items and the main focus was on general depressive symptoms and typical relationship problems the factor was named “*Depressive Personality.*” With those general characteristics of items being part of depression itself, all patients scored high on this factor with no meaningful variation (cp. **Table 3**).

**Table 1:** The 20 highest factor loadings on SWAP-200 items for Q-Factor 1 (“Depressive Personality”).

	20 highest factor loadings with SWAP-200 items on Factor 1	Factor loadings (*z*-values)
1	Tends to feel unhappy, depressed, or despondent	3.089
2	Tends to fear s/he will be rejected or abandoned by those who are emotionally significant	2.855
3	Tends to feel listless, fatigued, or lacking in energy	2.850
4	Tends to blame self or feel responsible for bad things that happen	2.774
5	Appears to find little or no pleasure, satisfaction, or enjoyment in life’s activities	2.417
6	Tends to feel s/he is inadequate, inferior, or a failure	2.245
7	Has difficulty acknowledging or expressing anger	2.034
8	Tends to be self-critical; sets unrealistically high standards for self and is intolerant of own human defects	2.012
9	Is simultaneously needy of, and rejecting toward, others (e.g., craves intimacy and caring, but tends to reject it when offered)	2.006
10	Tends to feel empty or bored	2.001
11	Tends to feel guilty	1.900
12	Tends to avoid confiding in others for fear of betrayal; expects things s/he says or does will be used against him/her	1.848
13	Tends to be insufficiently concerned with meeting own needs; appears not to feel entitled to get or ask for things s/he deserves	1.763
14	Tends to express aggression in passive and indirect ways (e.g., may make mistakes, procrastinate, forget, become sulky, etc.)	1.703
15	Tends to feel misunderstood, mistreated, or victimized	1.687
16	Tends to be overly needy or dependent; requires excessive reassurance or approval	1.683
17	Tends to feel s/he is not his/her true self with others; tends to feel false or fraudulent	1.661
18	Tends to be inhibited or constricted; has difficulty allowing self to acknowledge or express wishes and impulses	1.635
19	Tends to be critical of others	1.625
20	Tends to be anxious	1.625

In contrast, the 20 highest SWAP-200 items loading on Factor 2 (compare **Table 2**) seemed to be more specific for specific personality syndromes in depression and hence displayed greater variation and could better differentiate between subjects. Items of this factor broadly reflected characteristics that could be described as highly emotional, externally oriented (externalizing), and hostile. In various items emotions of high intensity (e.g., 1, 7) were evident in different contexts. Furthermore, many items described intense emotional interactions with others, pointing to an orientation toward the external world (as opposed to a social withdrawal evident in other types of depression). Those interactions could reflect dependency (e.g., 9, 15, 19) but mainly had a hostile or aggressive tone (e.g., 2, 5, 18, 20). In conjunction, subjects scoring high on this factor seemed to engage widely in interactions with others, typically in a hostile or dependent way with intensive emotions involved. This factor was therefore named after the dominant features “*Emotional-Hostile-Externalizing Personality*” resembling two subtypes of depression that have been described before (Shedler and Westen, 2004).

**Table 2:** The 20 highest factor loadings on SWAP-200 items for Q-Factor 2 (“Emotional-Hostile-Externalizing”).

	20 highest factor loadings with SWAP-200 Items on Factor 2	Factor loadings (*z*-values)
1	Tends to react to criticism with feelings of rage or humiliation	3.134
2	Tends to feel misunderstood, mistreated, or victimized	2.806
3	Tends to be emotionally intrusive; tends not to respect others’ needs for autonomy, privacy, etc.	2.487
4	Tends to think others are envious of him/her	2.436
5	Is quick to assume that others wish to harm or take advantage of him/her; tends to perceive malevolent intentions in others’ words and actions	2.397
6	Tends to blame others for own failures or shortcomings; tends to believe his/her problems are caused by external factors	2.262
7	Emotions tend to spiral out of control, leading to extremes of anxiety, sadness, rage, excitement, etc.	2.131
8	Tends to be competitive with others (whether consciously or unconsciously)	2.009
9	Tends to be overly needy or dependent; requires excessive reassurance or approval	1.670
10	Is preoccupied with the feeling that someone or something has been irretrievably lost (e.g., love, youth, the chance for happiness, etc.)	1.605
11	Tends to feel like an outcast or outsider; feels as if s/he does not truly belong	1.574
12	Tends to feel helpless, powerless, or at the mercy of forces outside his/her control	1.521
13	Tends to get into power struggles	1.479
14	Tends to hold grudges; may dwell on insults or slights for long periods	1.471
15	Tends to become attached quickly or intensely; develops feelings, expectations, etc. that are not warranted by the history or context of the relationship	1.416
16	Tends to feel envious	1.405
17	Has fantasies of unlimited success, power, beauty, talent, brilliance, etc.	1.372
18	Tends to be arrogant, haughty, or dismissive	1.330
19	Appears to fear being alone; may go to great lengths to avoid being alone	1.213
20	Tends to be angry or hostile (whether consciously or unconsciously)	1.161

**Table 3:** Factor scores of 20 patients on the factor “Depressive Personality” and “Emotional-Hostile-Externalizing Personality.”

	Factors	
	Depressive Personality	Emotional-Hostile-Externalizing
Patient 1	0.554	0.454
Patient 2	0.765	0.170
Patient 3	0.625	-0.213
Patient 4	0.782	-0.045
Patient 5	0.649	-0.488
Patient 6	0.791	-0.078
Patient 7	0.686	-0.060
Patient 8	0.628	-0.412
Patient 9	0.643	0.059
Patient 10	0.843	0.034
Patient 11	0.666	0.276
Patient 12	0.650	0.061
Patient 13	0.697	-0.296
Patient 14	0.649	-0.116
Patient 15	0.555	0.331
Patient 16	0.605	0.463
Patient 17	0.629	0.450
Patient 18	0.702	0.000
Patient 19	0.670	-0.368
Patient 20	0.762	-0.059

Factors 1 and 2 were uncorrelated (*r* = -0.27, *p* = 0.26)^1^.

### NEUROIMAGING RESULTS

**FIGURE 1:**
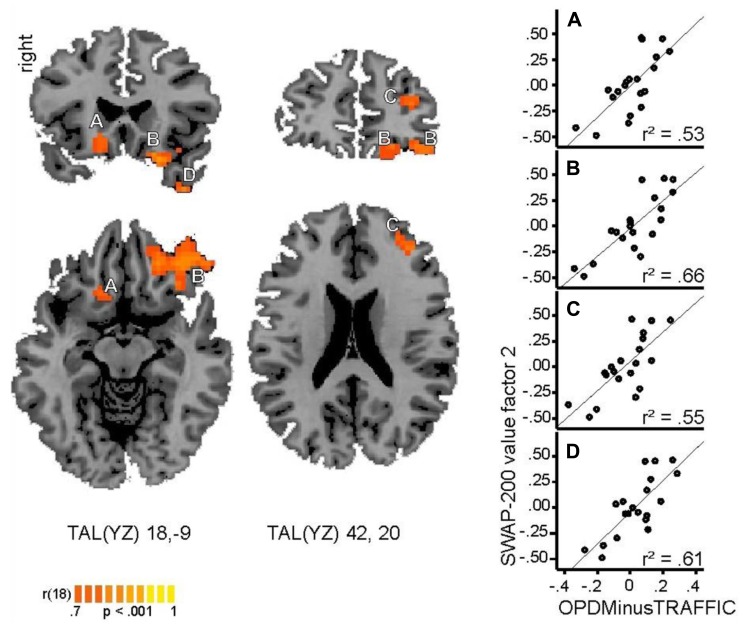
**Regions with significant positive correlation (*p* < 0.001, cluster size threshold of 16 voxels) between individual values within the SWAP-200 factor “Emotional-Hostile-Externalizing” and beta values for the contrast OPD > traffic.** Cluster A: ventral striatum (caudate head), B: orbitofrontal cortex, C: prefrontal cortex, D: temporal pole. Coordinates are provided in Talairach space. Right side: correlation coefficients between beta values within each cluster and SWAP-200 values for Factor 2.

The whole-brain correlational analysis yielded no significant correlation for Factor 1. Concerning Factor 2, four regions with significant positive correlations (*p* < 0.001, cluster size threshold of 16 voxels) between patients’ factor scores on the SWAP-200 factor “Emotional-Hostile-Externalizing Personality” and beta values for the contrast OPD > traffic could be identified: a large cluster in the right orbitofrontal cortex [OFC; anatomically within the inferior frontal gyrus (IFG)], the left ventral striatum (caudate head), a small cluster in the left temporal pole and another small cluster in the right middle frontal gyrus (functionally within the prefrontal cortex). See **Table 4** and **Figure 1** for details.

**Table 4:** Regions with significant positive correlation (*p* < 0.001, cluster size threshold of 16 voxels) between individual values within the SWAP-200 factor “Emotional-Hostile-Externalizing” and beta values for the contrast OPD > traffic.

Regions	Side	BA	Cluster size	*X*	*Y*	*Z*	*R*
Ventral striatum (caudate head)	R		594	13	18	-9	0.74
Inferior frontal gyrus (orbitofrontal cortex)	L	47,11	6507	-29	33	-12	0.84
Middle frontal gyrus	L	10	675	-33	42	20	0.77
Superior temporal gyrus (temporal pole)	L	38	567	-38	14	-36	0.81

## DISCUSSION

In the growing area of neuropsychoanalysis (Solms and Turnbull, 2011), this exploratory study can be described as “psychoanalytically informed neuroscience” that unifies an experimental design with a rich clinical assessment of patients with chronic depression to associate with brain activity. The present study brought together two issues regarding the heterogeneity in depression and analyzed brain data in an exploratory way. We used the SWAP-200 Q-Sort procedure to provide a clinically meaningful characterization of a sample of 20 chronically depressed patients and describe its correlations with brain activation using an individually tailored and depression-related paradigm. Twenty patients with chronic depression were confronted with their individual dysfunctional relationship pattern (derived from OPD) inside the fMRI scanner.

Entering the SWAP-200 items into a Q-factor analysis yielded two meaningful factors, “Depressive Personality” and “Emotional-Hostile-Externalizing Personality.” Only the second factor was differentiating between patients (high and low factor scores). Patients were distributed along this factor reflected by differences in emotion accompanied by relationship difficulties and hostile attributions toward others. In an exploratory analysis, values for both factors were correlated with beta values from the brain activity when patients were confronted with their dysfunctional relationship pattern (relative to a control condition). Interestingly, this whole-brain analysis yielded no correlations with the factor “Depressive Personality” and four distinct areas with Factor 2, whose activity significantly correlates with the extent to which patients are “Emotional-Hostile-Externalizing”: a large cluster in the right OFC (anatomically within the IFG), the left ventral striatum (caudate head), a small cluster in the left temporal pole, and another small cluster in the right middle frontal gyrus (functionally within the prefrontal cortex). Since this was an exploratory study with an open approach to analyses (Q-factor analysis and brain–behavior correlations) and no specific hypotheses, the discussion of the possible meaning and implication of our results is of course speculative in nature. Additionally, our results were correlational. Hence causal inferences could not be made and patients are distributed along a spectrum comprising the factor “Emotional-Hostile-Externalizing Personality” rather than forming a distinct subgroup. The fact, that – despite an open whole-brain approach – there were only four areas evident, of which three fit functionally into the framework of “Emotional-Hostile-Externalizing” (see below) encourages us to consider this study as hypothesis-generating. Future studies could chose subjects based on their characteristics in terms of “Emotional-Hostile-Externalizing Personality” (e.g., high vs. low) and conduct the fMRI experiment with *a priori* hypotheses to reject or confirm if the brain areas found here actually are involved differentially when processing a dysfunctional relationship pattern.

As for the regions, OFC, ventral striatum, and temporal pole are all part of the limbic system, broadly involved in emotion processing (Olson et al., 2007; Kopell and Greenberg, 2008). Generally speaking, activity in the limbic system in response to personally relevant emotional situations (OPD relationship pattern) that increases with clinically validated emotionality of the patient (“Emotional-Hostile-Externalizing”) is very plausible. In detail, OFC and ventral striatum together form a central part of the limbic loop in a recent model of basal ganglia functionality (Kopell and Greenberg, 2008). This limbic loop – as well as the OFC itself – is, amongst other functions, involved in emotion processing, the assessment of stimuli according to reward and punishment and reward based decision-making (Rolls, 2000). Interestingly, existing models differentiate between more lateral and more medial areas of the OFC providing different functions. In an early review (Kringelbach and Rolls, 2004), the authors argue for a relative specialization of the medial OFC in the processing of rewarding and the lateral OFC in the processing of punishing stimuli. The relative OFC activity in the current study is widespread but relatively more lateral and one characteristic of patients scoring high on the factor is their hostility toward others (with aspects like criticism, victimization, or grudge). Hence, the “punishing” aspect of dysfunctional relationship patterns presented in the fMRI could be relatively more important for patients with high hostile attributions. This “punishing” aspect is supposedly associated with a relatively lateral OFC activity.

On a more general level, the OFC is involved in emotional experiences and social behavior (Rolls et al., 1994; Hornak et al., 1996; Zald and Kim, 1996). This is interesting, since patients scoring high in the “Emotional-Hostile-Externalizing” factor display relatively greater involvement in social interactions (irrespective of valence) and have relatively more activity in the OFC.

Kircher et al. (2013) found the IFG (anatomical overlap with our OFC site) directly related to changes in symptom severity in panic disorder after a cognitive-behavioral psychotherapy. Before psychotherapy the relative stronger activation in the IFG in the group of patients was also related to a stronger connectivity between the IFG and the limbic system (amygdala, anterior insula, ACC). The authors tentatively speculated that specific cognitive processes in the IFG in terms of negative cognitions may trigger emotional processes. In our sample of chronically depressed patients, the relative higher activation in the OFC (anatomically IFG) may be related to stronger negative cognitions in patients scoring high in terms of hostile attributions toward others when being confronted with dysfunctional relationship patterns.

In itself, the ventral striatum is an area that has been repeatedly discussed in the pathophysiology of depression in several reviews and meta-analyses (Fitzgerald et al., 2008; Hamilton et al., 2012) and might also be a viable target for deep brain stimulation in otherwise treatment-resistant depression (Kopell and Greenberg, 2008). Hence, correlation between a clinical variable describing an aspect of depression and activity in the ventral striatum is very plausible.

The relationship-related aspects of patients scoring high on the factor “Emotional-Hostile-Externalizing” may also be related to the relative activation in the left temporal pole. This small region is also part of the limbic system and has been considered to be strongly involved in social and emotional processing (Olson et al., 2007). Additionally, the temporal pole – especially on the left side – is discussed as being a core area for tasks involving “mentalizing” (Frith and Frith, 2003). The basic idea is that the temporal pole processes access to social knowledge and social scripts. Receiving input from all sensory modalities and the other parts of the limbic system, the temporal pole is active when recalling autobiographical information, putting recent stimuli in the context of past experiences in social interactions (Frith and Frith, 2003). This function could be linked with the aspect of stronger conflicted relationships within the SWAP-200 factor “Emotional-Hostile-Externalizing” when thinking about their individual repetitive interaction patterns.

In summary, we found in an open whole-brain correlation analysis that the degree of patients with depression to react with intense emotions, engage heavily in social interactions and tend to be or view their environment as hostile (SWAP-200 factor “Emotional-Hostile-Externalizing”) correlated positively with relatively higher activity in three key areas involved in emotion processing, evaluation of reward/punishment, depressive pathology, negative cognitions, and social knowledge (OFC, ventral striatum, temporal pole). We speculate here, that those patients scoring higher in “Emotional-Hostile-Externalizing” reacted with stronger emotions when confronted with their dysfunctional relationship pattern, had a tendency to evaluate the stimuli as being more punishing or experienced stronger negative cognitions and engaged more intensively in the recall of social situations. Results may contribute to an alternative description of neural correlates of depression showing differential brain activation dependent on personality syndrome related subtypes of depression. Future studies should include other patient groups, e.g., anxiety disorders, to analyze whether the results reported here are specific to depression or have an overlap to other mental disorders.

## Conflict of Interest Statement

The authors declare that the research was conducted in the absence of any commercial or financial relationships that could be construed as a potential conflict of interest.

## AUTHOR CONTRIBUTIONS

Daniel Wiswede, Henrik Kessler, and Svenja Taubner have conducted the study. Daniel Wiswede was mainly responsible for fMRI-data acquisition and data analysis. Henrik Kessler and Svenja Taubner were responsible for participant recruitment, clinical assessments, and data analysis. Svenja Taubner assessed the SWAP-data and had the basic idea for this manuscript. All authors had full access to all of the data in the study and take responsibility for the integrity of the data and the accuracy of the data analysis. All authors contributed in writing the manuscript.

## Footnotes

1 We tested another principle component analysis using varimax rotation to compare results. This also led to a two-factor solution but patients’ factor scores were highly correlated in this sample (*r* $=$ 0.89) which confirmed our theoretical assumption to avoid a simple structure approach with this material.">We tested another principle component analysis using varimax rotation to compare results. This also led to a two-factor solution but patients’ factor scores were highly correlated in this sample (*r* $=$ 0.89) which confirmed our theoretical assumption to avoid a simple structure approach with this material.
